# Editorial: Immunologic tumor microenvironment modulators for turning “cold” tumors to “hot” tumors

**DOI:** 10.3389/fimmu.2024.1425136

**Published:** 2024-05-15

**Authors:** Mazdak Ganjalıkhani-Hakemi, Gulderen Yanikkaya Demirel, Xin He, Chengwu Zeng

**Affiliations:** ^1^ Department of Immunology, Faculty of Medicine, Isfahan University of Medical Sciences, Isfahan, Iran; ^2^ Regenerative and Restorative Medicine Research Center (REMER), Research Institute for Health Sciences and Technologies (SABITA), Istanbul Medipol University, Istanbul, Türkiye; ^3^ Department of Immunology, Faculty of Medicine, Yeditepe University, Istanbul, Türkiye; ^4^ Department of Hematological Malignancies Translational Science, Gehr Family Center for Leukemia Research, Hematologic Malignancies and Stem Cell Transplantation Institute, Beckman Research Institute, City of Hope Medical Center, Duarte, CA, United States; ^5^ Department of Hematology, The Fifth Affiliated Hospital of Guangzhou Medical University, Guangzhou, China

**Keywords:** cold tumor, small peptide, small molecule, bioactive peptide, cancer vaccine, immune check-point inhibitor

Cancer immunotherapy harnesses the body’s immune system to combat tumors while sparing normal cells. Numerous strategies have been explored for this purpose. However, monotherapy using these methods often proves ineffective in clinical trials. Many tumors resist immunotherapy, earning them the designation of “cold” or non-inflammatory tumors. These cold tumors lack sufficient infiltration by CD8+ T cells, hampering immune response. They are characterized by a dearth of cytotoxic T cells, alongside the presence of anti-inflammatory myeloid cells, tumor-associated M2 macrophages, and regulatory T cells. Combining immunotherapy with other cancer treatment modalities, such as chemotherapy or cancer vaccines, holds promise in bolstering efficacy and improving outcomes (1–3).

In their article titled “Overcoming cold tumors: a combination strategy of immune checkpoint inhibitors,” Ouyang et al. have explored methods to convert cold tumors into hot ones, including boosting T cell infiltration and adopting therapies like CAR T cells. Despite the groundbreaking impact of Immune Checkpoint Inhibitors (ICIs) on cancer therapy, resistance persists in many cold tumors due to diverse immune evasion mechanisms. The success of immunotherapy hinges on T cells’ capacity to recognize and eliminate tumor cells; however, cold tumors lack T cell infiltration, rendering ICI therapy ineffective. Overcoming these challenges, particularly impaired T cell activation and homing, is essential for enhancing ICI therapy efficacy.

In one of the articles within this Research Topic, titled “Optimal combination of MYCN differential gene and cellular senescence gene predicts adverse outcomes in patients with neuroblastoma,” the focus was on predicting neuroblastoma (NB) prognosis. Utilizing a predictive signature based on six optimal candidate genes (TP53, IL-7, PDGFRA, S100B, DLL3, and TP63), the study demonstrates superior prognostic capability compared to an individual gene analysis. The signature also sheds light on the immunosuppressive and aging tumor microenvironment in MYCN-amplified high-risk NB patients.

“Cytotoxic response of tumor-infiltrating lymphocytes of head and neck cancer slice cultures under mitochondrial dysfunction” by Greier et al. is about head and neck squamous cell carcinomas (HNSCC). They have cultivated slice cultures of the HNSCC to test the effect of mitochondrial dysfunction on cytotoxic T cell under different metabolic conditions. They have found that high glucose concentration alone did not have any impact on T cell activity or apoptosis while mitochondrial dysfunction with alone increased the apoptosis in tumor cells.

An article by Cini et al., is about a novel fusion protein SON-1210 (IL-12-FHAB-IL-15) produced with anticipation to amplify the therapeutic impact of interleukins and combination immunotherapies in human tumor microenvironment (TME). SON-1210 is a fused single-chain human IL-12 and native human IL-15 in cis onto a fully human albumin binding (FHAB) domain single-chain antibody fragment (scFv). They have shared the results of their experiments *in vitro* and in animal models on cytotoxicity, pharmacokinetics, potency, functional characteristics, safety, immune response, and efficacy. The authors suggest that linking cytokines to a fully human albumin-binding domain provides an indirect opportunity to target the TME using potent cytokines in cis that can redirect the immune response and control tumor growth.

Same group of researchers have also shared their Phase I trial results with SON-1210 in another article, and declared that SON-1010, a novel presentation for rIL-12, was safe and well tolerated in healthy volunteers up to 300 ng/kg. They emphasize that extended half-life of the drug leads to a prolonged and controlled IFNγ response, which may be important for tumor control in patients.

Despite some successes in immunotherapy for oncological diseases, cold tumors pose a significant therapeutic challenge. It is anticipated that future treatment algorithms will adapt therapeutic strategies to the immune context of tumors, as treatment with checkpoint inhibitors or vaccines alone often falls short. Therefore, combining other therapeutic approaches with existing methods may prove more effective for cold tumors, which either weakly stimulate or resist the immune system (4, 5).

Tong et. al., in their “Making “cold” tumors “hot”-Radiotherapy remodels the tumor immune microenvironment of pancreatic cancer to benefit from Immunotherapy: A case report” titled article, reported a case of advanced metastatic cancer treated with immunotherapy combined with chemotherapy and radiotherapy where they have observed a sharp shift of TIME from T3 to T2. They propose that this combination may have significant therapeutic benefits suggesting a new strategy for the treatment of advanced pancreatic cancers.

Shi et. al., in their review article “Neoadjuvant SBRT combined with immunotherapy in NSCLC: from mechanisms to therapy”, have provided updated information on use of Stereotactic Body Radiotherapy (SBRT) inducing direct tumor cell death and stimulation for local and systemic anti-tumor immune responses for early stage resectable non-small-cell lung cancers (NSCLC). They have provided information about the clinical trials combining the immunotherapy and SBRT after surgical resection and also discussed the optimal dosage, therapy schedule and biomarkers to be used in clinical applications.

The article “It’s high-time to re-evaluate the value of induced-chemotherapy for reinforcing immunotherapy in colorectal cancer” underscores the importance of induced chemotherapy in enhancing immunotherapy for colorectal cancer (CRC). Certain chemotherapeutic agents exhibit immune-stimulatory properties, such as inducing immunogenic cell death (ICD) and promoting the generation of non-mutated neoantigens (NM-neoAgs). Despite the remaining challenges, clinical trials have shown promise for this combination approach in improving immunotherapy efficacy in CRC.

Wang et al., in their review titled “Utilizing Exosomes as Sparking Clinical Biomarkers and Therapeutic Response in acute myeloid leukemia,” comprehensively outline advancements in understanding the involvement of exosomes in AML pathogenesis. This synthesis is pivotal for advancing the utilization of exosomes in both diagnosis and treatment strategies for AML.

In another work titled “Targeting LSD1 in Tumor Immunotherapy: Rationale, Challenges, and Prospects,” Bao et al. succinctly encapsulated recent progress in the intersection of LSD1 and tumor immunity, proposing a potential therapeutic avenue by integrating LSD1 inhibition with immunotherapy protocols.

While CAR T cell therapy shows promise in hematological cancers, its efficacy in solid tumors like pancreatic cancer is limited by the immunosuppressive tumor microenvironment (TME). Akbari et al. consider the role of Prostaglandin E2 (PGE2) in their article entitled “PGE2-EP2/EP4 signaling elicits mesoCAR T cell immunosuppression in pancreatic cancer.” Their investigations reveal a negative correlation between PGE2 expression and memory T cell gene signatures in pancreatic cancer tissue. They conclude that blocking PGE2-EP2/EP4 signaling may enhance CAR T cell activity in this challenging TME.

Additionally, Meymandi et al., in their work entitled “PX-478, an HIF-1a inhibitor, impairs mesoCAR T cell antitumor function in cervical cancer,” consider hypoxia’s impact on HIF-1a expression and CAR T cell therapy’s low success rate in solid tumors like cervical cancer. Their experiments demonstrate that PX-478 inhibits T cell proliferation, impairs cytotoxicity, and induces exhaustion, highlighting the relevance of HIF-1a in T and CAR T cell function.

To combat immunosuppressive TMEs, targeted treatments utilizing small molecules, peptides, or other materials capable of disrupting the TME can be employed as adjuvant therapies. Ghadiri et al. reviewed bioactive peptides from plant and animal sources in their article “Bioactive peptides: an alternative therapeutic approach for cancer management.” These peptides have shown promise in inhibiting cancer cell proliferation, inducing apoptosis, and suppressing tumor growth and metastasis.

This Research Topic covers advances in immunology, medical chemistry, biochemistry, pharmacology, food engineering, and molecular biology relevant to cancer treatment. Out of 26 articles received, 14 were accepted for publication, including 8 reviews, 5 original articles, 1 clinical trial, and 1 case report. These contributions paved the way toward new research directions related to immunologic tumor microenvironment modulators, aiming to convert cold tumors into hot ones. It is hoped that these efforts and the articles presented in this Research Topic will be interesting, informative, and inspiring to readers, encouraging further exploration of this important subject.

## Author contributions

MG-H: Writing – review & editing, Writing – original draft, Supervision, Conceptualization. GYD: Writing – review & editing, Writing – original draft. XH: Writing – review & editing, Writing – original draft. CZ: Writing – review & editing, Writing – original draft.
